# Acute eosinophilic pneumonia associated with smoking: a case report

**DOI:** 10.1080/20009666.2018.1466601

**Published:** 2018-06-12

**Authors:** Beenish Fayyaz

**Affiliations:** Greater Baltimore Medical Center, Towson, MD, USA

**Keywords:** Acute eosinophilic pneumonia, peripheral eosinophilia, bronchoalveolar lavage, community-acquired pneumonia, tobacco smoking, cortcicosteroids

## Abstract

Acute eosinophilic pneumonia (AEP) is commonly misdiagnosed as infectious pneumonia due to presence of fever and radiological features. However, development of peripheral eosinophilia within days of presentation should raise the concern of AEP especially in previously heathy adults with history of recent tobacco smoking.

## 1. Introduction

Acute eosinophilic pneumonia is categorized within the heterogenous group of eosinophilic lung diseases and is associated with airway and/or lung tissue eosinophilia in the absence of other causes of eosinophilia such as vasculitis and fungal/parasitic infections. AEP is sometimes classified as ‘idiopathic’ AEP (IAEP) as no cause can be identified in most of the cases. However, it has recently been suggested that these idiopathic cases might be linked to tobacco smoking although the exact pathophysiology is still unknown.

We present a case when a young adult was initially admitted with a presumptive diagnosis of community-acquired pneumonia (CAP) but was determined to have AEP in the context of recent tobacco smoking.

## 2. Case description

**Figure 1.**
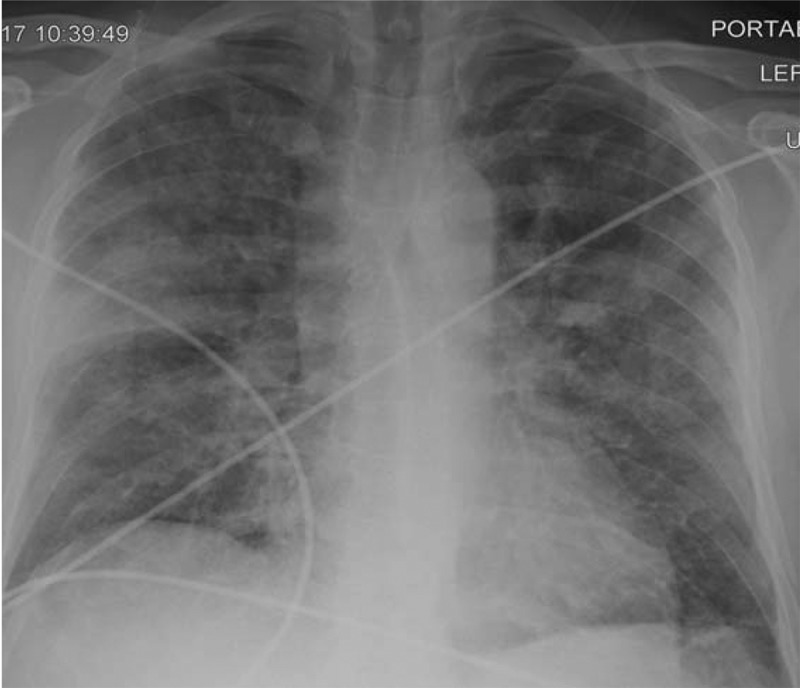
Chest radiograph on admission demonstrating bilateral diffuse multifocal infiltrates, most pronounced on the right side.

**Figure 2.**
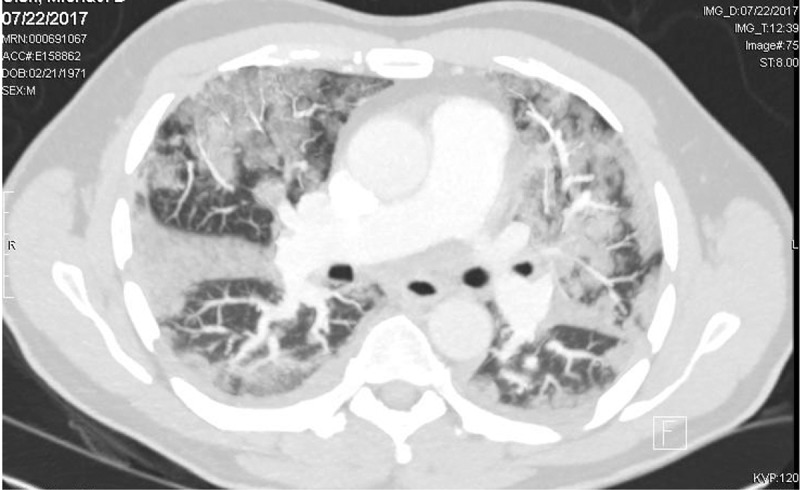
CT Chest shows multifocal patchy airspace opacities and small pleural effusions.

A previously healthy 46-year-old man presented to emergency department with a 2-day history of cough and high-grade fever. On the morning of presentation, he had developed sudden onset shortness of breath while at rest. He had a history of cigarette smoking for 4 months before the development of symptoms. He initially smoked two to three cigarettes on weekends when with friends but had been smoking three to four cigarettes on a daily basis for the last few weeks. He had no previous history of asthma, atopy or illicit drug use. He denied being exposed to any animals, birds or livestock. He had not travelled during the last one year and denied being exposed to chemical fumes. On admission, vitals were as follows: blood pressure of 110/88 mm Hg, pulse of 100/min and respiratory rate of 18/min. He was noted to have SpO2 of 75% on room air which improved to 91% on 10 L of oxygen via nasal cannula. Auscultation of the lungs revealed bilateral coarse crackles. The rest of the examination was unremarkable, and no rash, lymphadenopathy and pallor were noted. His peripheral white blood cell counts were 8500/μL with 78% neutrophils and 4% eosinophils. Serum C-reactive protein (CRP) was 32.36 mg/dL, pro-calcitonin was 0.70 ng/mL and erythrocyte sedimentation rate was 64 mm/hour. Renal parameters and urinalysis were unremarkable. Chest radiograph showed diffuse bilateral infiltrates (Figure 1). Chest computed tomography showed patchy bilateral opacifications and bilateral pleural effusions (Figure 2).

He was started on IV antibiotics, ceftriaxone and azithromycin, for the treatment of presumed CAP. Oral prednisone 50 mg once daily was also added to his regimen considering that he had a severe form of CAP based on his hypoxemia and elevated CRP. By hospital day 2, he reported feeling much better along with lower oxygen requirements. Infectious workup for pneumonia such as *Legionella* antigen, *Streptococcus* antigen and blood cultures were unremarkable.

**Figure 3.**
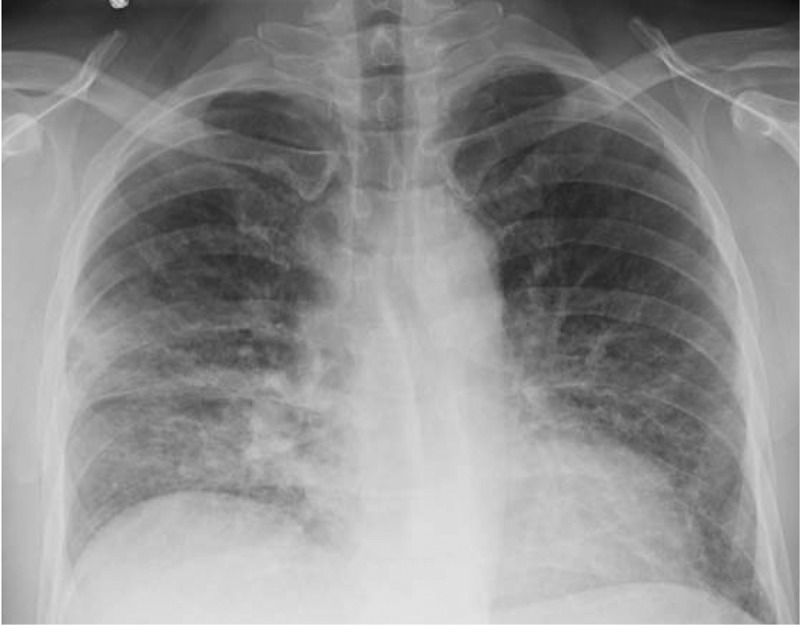
Chest radiograph done on day 6 shows improvement in bilateral infiltrates.

By hospital day 3, he was noted to have mildly elevated eosinophil count of 9% which continued to rise reaching up to 35% by day 6. He continued to be clinically stable despite having a persistent dry cough while repeat chest radiograph showed significant improvement (Figure 3). He was found to have normal IgE levels while workup for auto-immune antibodies (ANA, ANCA), hypersensitivity pneumonitis panel and fungal infection (beta-D-glucan, *Aspergillus, Coccidioides*) was also negative. Echocardiogram done at this point showed normal left ventricular function with ejection fraction (EF) of 50% and normal estimated pulmonary artery pressure.

**Figure 4.**
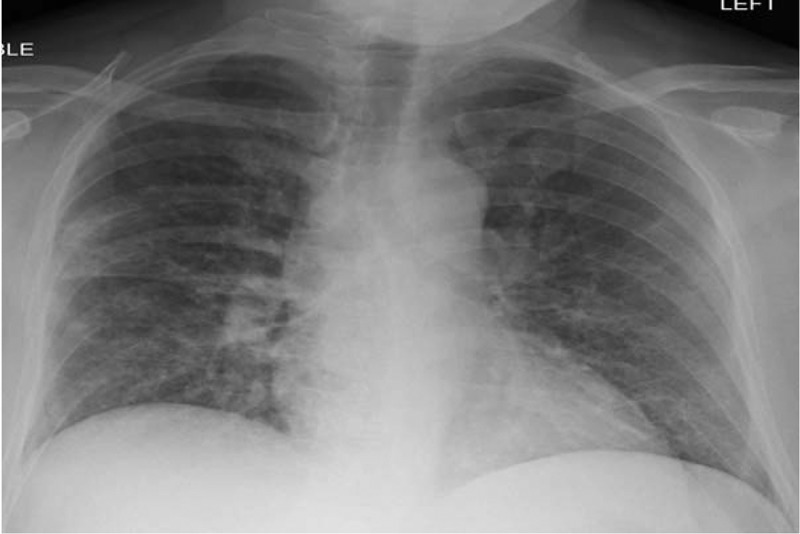
Chest radiograph done after discharge showed resolution of pulmonary infiltrates.

Although the patient was improving, bronchoscopy was performed to evaluate for an underlying eosinophilic lung disorder due to persistent dry cough in the setting of peripheral eosinophilia. Bronchoalveolar lavage (BAL) fluid cytology showed an eosinophil count of 12% while bronchial cultures (fungal and bacterial) were negative. Based on these findings, the patient was diagnosed as having AEP. Due to concerns of drug (ceftriaxone)-induced AEP, antibiotics were discontinued by day 8. However, eosinophil count had already started to improve (26%) before this step was taken. Therefore, it was determined that this episode of AEP was likely due to his recent cigarette smoking. He was discharged on a corticosteroid taper and advised to follow as outpatient. He continued to remain asymptomatic while refraining from smoking. Chest radiograph done as outpatient showed resolution of the infiltrates (Figure 4).

It is important to mention here that other lung disorders like eosinophilic granulomatosis with polyangitis can cause pulmonary infiltrates along with pulmonary eosinophilia but it was considered unlikely in our patient in the absence of other organ dysfunction and autoimmune workup was also unremarkable. In addition, he has continued to do well after the rapid steroid taper with no recurrence of pulmonary infiltrates for the last 18 months.

## 3. Discussion

AEP is a serious and rare form of pulmonary eosinophilia which was first distinctly identified in 1989 by Allen et al. [1]. It is characterized as a febrile illness associated with acute onset respiratory failure, bilateral diffuse pulmonary infiltrates on imaging and eosinophils >25% on BAL or lung biopsy. All other causes of pulmonary eosinophilia need to be ruled out before AEP is diagnosed [2].

AEP can be caused by various parasitic infections and drugs but is idiopathic in most of the cases. What makes our case report interesting is that in the past few years, the so-called IAEP has been found to be strongly associated with tobacco smoke exposure which can be recent initiation of smoking, change in smoking habits such as re-introduction or even short-term passive smoking [3]. The pathogenesis of smoking induced AEP is not well understood till now although it is hypothesized to occur due to respiratory epithelial injury with subsequent eosinophil recruitment, degranulation and inflammation [2]. We believe that AEP was secondary to cigarette use in our patient who had recently started smoking.

Due to similarity in symptoms and radiological findings, AEP can be misdiagnosed as CAP or ARDS initially. Chest imaging in AEP demonstrates diffuse infiltrates/opacities, bilateral pleural effusion and interstitial edema [4]. CT Chest done for our patient showed similar findings (Figure 2) but were presumed to be due to severe/atypical CAP and thus, treated with IV antibiotics. Interestingly, he was also started on oral corticosteroids as adjuvant therapy based on recent data showing benefit in patients with severe CAP [5]. This intervention was likely responsible for clinical improvement in our patient as AEP is also treated with corticosteroids.

Although AEP is a type of eosinophilic disorder, presence of peripheral eosinophilia is not required for the diagnosis of AEP. This is because most of the time, the peripheral eosinophilia may be absent or late-onset. This is especially true in cases associated with cigarette smoking [6]. The same scenario was observed in our patient who developed peripheral eosinophilia on the third day of admission which raised the concern for an eosinophilic lung disease and thus evaluated via a bronchoscopy and BAL.

Eosinophil count >25% on BAL is one of the criterion required to diagnose AEP. Our patient had eosinophil count of 12% on his BAL. However, we feel this does not necessarily go against the diagnosis as BAL was performed on the 7th day of admission when he had already been on corticosteroids which might have led to a decreased eosinophilic burden. Similar cases have been documented in literature which were still treated as AEP as they fulfilled the other criteria such as fever, acute onset hypoxemia and diffuse pulmonary infiltrates [7]. A lung biopsy demonstrating eosinophils should confirm the diagnosis but unfortunately, was not done for our patient.

It can be argued that antibiotics may have led to initial clinical improvement instead of the steroids but then that does not explain the development of eosinophilia in our patient as bacterial pneumonias are not known to cause peripheral or pulmonary eosinophilia. In addition, antibiotic-induced AEP was also considered in our patient as peripheral eosinophilia developed after admission and improved after withdrawal of antibiotics. However, we feel this is less likely due to the following reasons: (1) antibiotics including ceftriaxone were started on admission thus not explaining the presenting symptoms and initial radiological findings. (2) Patient continued to improve clinically despite being on antibiotics and worsening peripheral eosinophilia. If the antibiotics were the cause, then the pulmonary infiltrates should not have resolved or at least should have recurred. This clinical improvement was likely due to concomitant corticosteroid treatment leading to decreased oxygen requirements and resolution of pulmonary infiltrates on chest X-ray. (3) Peripheral eosinophil counts in AEP fluctuate and do not correlate with clinical improvement. This is based on various case studies. Rhee et al. [8] noted that peripheral eosinophilia may improve initially but can worsen later during the disease despite adequate management and clinical response. On the other hand, Jhun et al. [9] noted that peripheral eosinophilia in milder cases of AEP may subside even without any treatment. In our patient, peripheral eosinophilia started improving even before withdrawal of antibiotics, thus not confirmative of drug-induced AEP.

## 4. Conclusion

AEP is a type of eosinophilic lung disease which presents with acute respiratory failure and has an excellent prognosis if treated appropriately with corticosteroids. The challenge lies in the timely diagnosis of this clinical entity due to its resemblance with infectious pneumonia or ARDS. Recent tobacco smoke exposure and peripheral eosinophilia are important clinical indicators suggestive of AEP.
